# Supplementary material for: Establishment of Primary Adult Skin Fibroblast Cell Lines from African Savanna Elephants (Loxodonta africana)
Source: Animals (Basel). 2023 Jul 19;13(14):2353. doi: 10.3390/ani13142353 (PMC10376752; doi:10.3390/ani13142353)
Supplement: Supplementary file 1 [file animals-13-02353-s001.zip › animals-2476720-supplementary.pdf]

Table S1: Cell culture and metaphase spread reagents and consumables.

Reagent/Consumable	Storage	Comments	Vendor
**Cell Culture reagents and consumables**			
Thianil®	Room temperature	Dosage/body mass: 3 mg/1 000 kg<br>Chemical composition: 10 mg thiafentanil oxalate and 0.1% methylparaben	Wildlife Pharmaceuticals PTY Ltd., Mpumlanaga, South Africa
Eagle's Minimum Essential Medium (EMEM)	2 – 8°C	Contains Earle's Balanced Salt Solution and L – Glutamine<br>Sterile	Lonza, Walkersville, Maryland, USA
Fetal Bovine Serum (FBS)	-20°C	cEMEM contained 20% FBS<br>Sterile	Gibco, Dun Laohaire, Dublin, UK
Phosphate Buffered Saline (PBS)	2 – 8°C	Sterile	Gibco, Dun Laohaire, Dublin, UK
Dimethyl Sulfoxide (DMSO)	Room temperature	Freezing media contained 90% FBS and 10% DMSO<br>Sterile	Sigma-Aldrich Co. LLC, St. Louis, Missouri, USA
Trypsin EDTA solution (1X)	Working solution: 2 – 8°C<br>Stock: -20°C	1 mL trypsin EDTA solution 1X was used to detach the fibroblasts from a T25 flask<br>Sterile	Lonza, Walkersville, Maryland, USA
Penicillin/Streptomycin/Amphotericin B (P/S/A)<br><br>Gentamycin	Working solution: 2 – 8°C<br>Stock solution: -20°C<br>Room temperature	Transport media contained 10% P/S/A<br>Washing PBS contained 10% P/S/A and 2% Gentamicin<br>Initial culturing media contained 20% FBS and 1% P/S/A<br>cEMEM contained 1% P/S	Lonza, Walkersville, Maryland, USA

Tissue cultured treated flasks (T25)<br>Conical centrifuge tubes (15 mL)	Room temperature	Sterile	Nest, Biotechnology Co., Ltd., Wuxi, China
Scalpels and surgical blades (size11)	Room temperature	Sterile	Hi-care Int, Cape Town, South Africa
Glass Pasteur pipets (150 mm)	Room temperature	Marien field laboratory glassware, unplugged<br>Sterile	Lasec, Am Wollerspfad, Lauda-Koningshofen, Germany
Glass petri dish (100 mm)	Room temperature	Sterile	Pyrex, England
Serological pipets (2 and 10 mL)	Room temperature	Sterile	Nest, Biotechnology Co., Ltd., Wuxi, China
Tweezer	Room temperature	Sterile	N/A

<u>Metaphase Spread reagents and consumables</u>

KaryoMAX™ Colcemid™	2 – 8 °C	Working solution: 10 μg/mL in PBS<br>0.05 μg/mL /petri dish	Gibco, Dun Laohaire, Dublin, UK
Tissue cultured treated petri dish (35 mm x 12 mm)	Room temperature	Sterile	Nest, Biotechnology Co., Ltd., Wuxi, China
Trypsin EDTA solution (1X)	Working solution: 2 – 8°C<br>Stock stored: -20 °C	0.5 mL trypsin EDTA solution 1X was used to detach the EDFs from a 35 mm petri dish<br>Sterile	Lonza, Walkersville, Maryland, USA
Potassium Chloride (KCl)	Powder: room temperature	5.6 g KCl powder was dissolved in 1 L dH_20, (0.075 M)	Sigma-Aldrich Co. LLC, St.

	Working solution: 2 – 8°C		Louis, Missouri, USA
Methanol/Acetic acid solution (3/1)	Working solution: room temperature	Cold fixative is not recommended in order to avoid the incorporation of humidity into the cell pellet due to condensation because water will disturb the chromosome spreading	Merck, Billercia, Darmstadt, Germany
Romanowsky- Giemsa stain (R – G) staining	Stock solution: room temperature	Solution is protected from light and stable for approximately 1 hour	Gurr®, BDH chemicals LTD., Poole, UK
HEPES (4 – (2 – hydroxyethyl) – 1 – piperazineethanesulfonic acid)	Room temperature	HEPES buffer stock solution: 900 mL solution A + 100 mL solution B (0.1 M, pH 6.5)<br><br>A: Solution A (HEPES sodium salt, $C_8H_{17}N_2NaO_4S$, Molecular Weight: 260.28): 23,83 g dissolved in 1 L dH_20<br>B: Solution B (HEPES, $C_8H_{18}N_2O_4S$, Molecular Weight: 238.30): 26 g dissolved in 1 L dH_20<br><br>HEPES buffer working solution: 300 mL HEPES buffer stock solution to 700 mL dH_2O (0.03 M, pH6.5)	Santa Cruz Biotechnology Inc., Dallas, Texas, USA
DPX mounting for histology	Room temperature	Mixture of distyrene, a plasticizer, and xylene	Kreatech Biotechnology, Villerweg, Amsterdam, Netherlands
Glass slides &<br>Glass coverslips	Room temperature	Glass slides were cleaned in methanol	Lasec, Am Wollerspfad, Lauda-

			Koningshofen, Germany
